# Case Report: A rare small bowel sarcoma

**DOI:** 10.3389/fsurg.2025.1456485

**Published:** 2025-03-11

**Authors:** Jiwu Guo, Jie Mao

**Affiliations:** Department of General Surgery, The Second Hospital & Clinical Medical School, Lanzhou University, Lanzhou, China

**Keywords:** small bowel sarcoma, hepatocellular cancer, immune checkpoint inhibitors, immune-related adverse reactions, case report

## Abstract

Intestinal sarcomas are rare gastrointestinal tumors but their etiology is not clear. A middle-aged man was admitted to hospital on 11 April 2023 because of intermittent melena for 1 month. Gastroscopy and colonoscopy were inconclusive, however, melena persisted. Abdominal magnetic resonance imaging indicated a left mid-abdominal mass and lymphoma of the small bowel origin. Small bowel enteroscopy revealed infiltrating periannular ulcer lesions in the jejunum. He underwent an operation and a partial resection of the small intestine was performed. The pathological examination revealed mesenchymal, highly malignant, poorly differentiated sarcoma. The patient died 6 months after surgery. The patient had been diagnosed with hepatocellular cancer and received immune checkpoint inhibitors (PD-1, sintilimab) combined with bevacizumab for 14 cycles 19 months before being diagnosed with intestinal sarcoma, and achieved complete remission. While immunotherapy achieves good therapeutic effects, another problem that has to be paid attention to is immune-related adverse reactions, which involve multiple systems. Based on the above information, we believe that this small bowel sarcoma was a rare complication of immunotherapy.

## Introduction

Small bowel neoplasms are rare, accounting for 3% of all gastrointestinal tumors, while intestinal sarcomas are unusual, accounting for 10%–15% of all small-bowel tumors (1). The main treatment for intestinal sarcoma is surgical resection; however, recurrence and metastasis after surgery affect the prognosis. In recent years, immunotherapy has achieved promising results in most tumors, but the results in sarcomas remain poor (2). Data from a patient with a small bowel sarcoma who received immunotherapy for hepatocellular cancer (HCC) were summarized and reported in this case report to alert clinicians to immune-related adverse events.

## Case presentation

**Figure 1:**
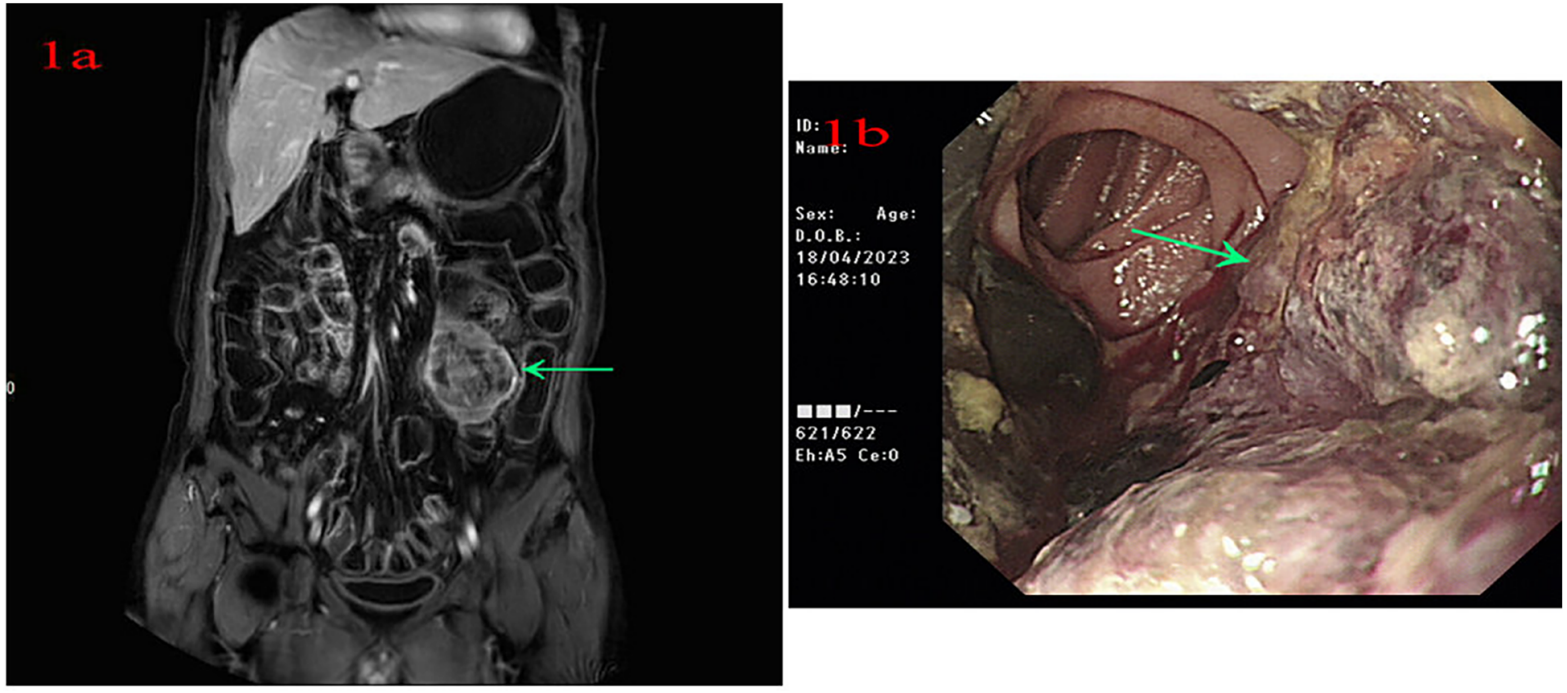
**(a)** Abdominal magnetic resonance imaging indicates a left mid-abdominal mass. **(b)** Small bowel enteroscopy shows infiltrating lesions of periannular ulcer at the jejunum.

**Figure 2:**
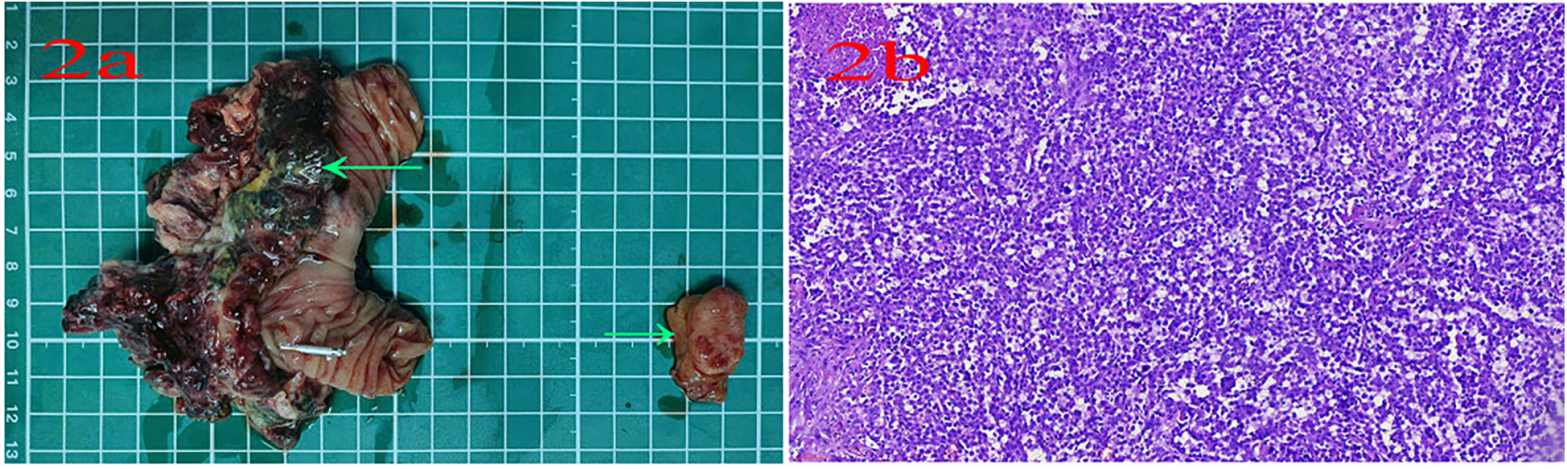
**(a)** Small bowel tumors. **(b)** Pathological examination for tumor.

A 56-year-old male patient was admitted to Lanzhou University Second Hospital on 11 April 2023 because of intermittent melena for 1 month. The patient had visited a local clinic. Gastroscopy and colonoscopy were inconclusive and the patient received a blood transfusion. However, melena persisted; therefore, the patient was admitted to the Lanzhou University Second Hospital. The patient had no history of diabetes. A physical examination revealed a heart rate of 85 bpm, blood pressure of 125/77 mmHg, respiration rate of 16 bpm, and pale palpebral conjunctiva. A physical examination of the abdomen showed no abnormalities. The laboratory test results were as follows: white blood cells, 10.10 × 10^9^/L; red blood cells, 3.36 × 10^12^/L; hemoglobin, 93 g/L; and platelets, 279 × 10^9^/L. Tests for anti-HBs and anti-HBc were positive. Abdominal magnetic resonance imaging (MRI) indicated a left mid-abdominal mass and lymphoma with small bowel origin. Extraintestinal tumors or abdominal metastases were suspected (Figure 1a). Small bowel enteroscopy revealed infiltrating periannular ulcer lesions in the jejunum (Figure 1b). On 20 April 2023, surgery was performed, and an 8 cm intestinal tumor was found 50 cm away from the suspensory ligament of Treitz; another tumor (2 cm × 2 cm) was observed 130 cm away from the suspensory ligament of Treitz. A partial resection of the small intestine was performed (Figure 2a). The pathological examination revealed a mesenchymal, highly malignant, poorly differentiated sarcoma (Figure 2b). The patient was discharged 6 days postoperatively. After surgery, the patient received an anlotinib hydrochloride capsule (12 mg qd) orally for two cycles, after which a liver metastatic tumor was diagnosed, and peritoneal metastasis was also found subsequently. The patient died 6 months after surgery.

**Figure 3:**
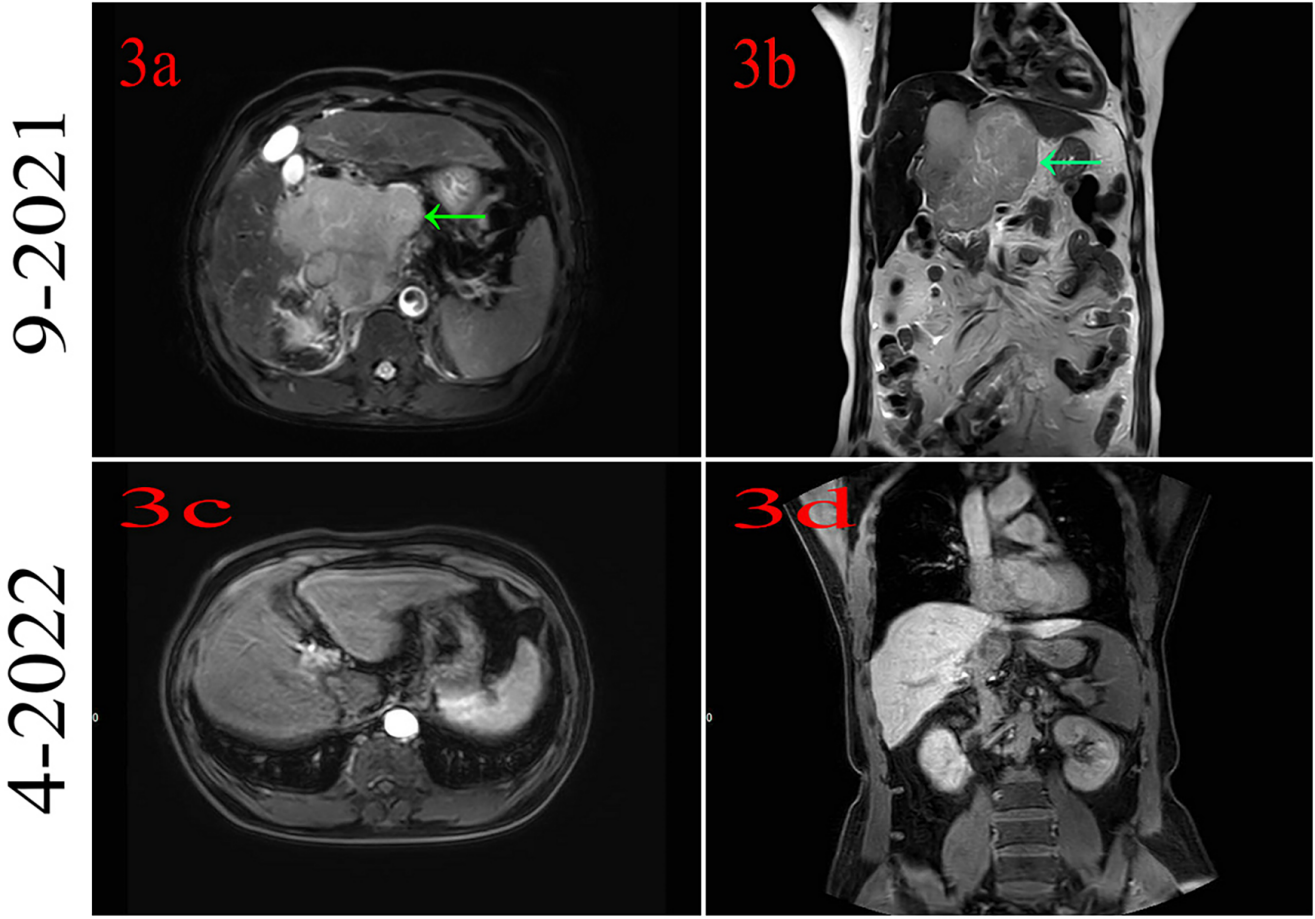
**(a,b)** Magnetic resonance imaging indicates hepatocellular cancer at segment 1. **(c,d)** MRI indicates that the patient had complete remission.

The patient had no family history of cancer or infectious disease but had a history of long-term alcohol consumption. In September 2021, the patient underwent a physical examination, and solid lesions were found in his liver. Subsequently, a laboratory examination found abnormal levels of alpha-fetoprotein (AFB) (9.50 ng/ml; normal: 0–8.78 ng/ml) and protein induced by vitamin K absence or antagonist-II (PIVKA II) (261.29 mAU/ml; normal: 13.62–40.38 mAU/ml). Gadoxetic acid disodium MRI indicated a huge mass in the S1 segment and cancer emboli were found in the right branch of the portal vein and the inferior vena cava (Figures 3a,b). Based on the above information, the patient was diagnosed with HCC and received systemic therapy with PD-1 (sintilimab 200 mg) combined with bevacizumab (600 mg), q3w. The patient achieved complete remission, and the medication was discontinued after 14 cycles (Figures 3c,d).

## Discussion

In 2020, a breakthrough was made in the treatment of liver cancer. Atezolizumab combined with bevacizumab has increased overall survival and progression-free survival in unresectable HCC patients (3). Subsequently, this regimen has been recommended by a series of authoritative guidelines and was rapidly promoted in clinical practice. Systemic therapy plays a leading role in advanced HCC treatment, and immune checkpoint inhibitors (ICIs) combined with bevacizumab or lenvatinib is the main therapeutic regimen for HCC that significantly prolongs overall survival (4). The patient received ICI combined with bevacizumab and achieved a satisfactory clinical outcome.

While immunotherapy has achieved good therapeutic effects, another problem that needs to be paid attention to is immune-related adverse reactions. Immune-related adverse events involve multiple systems, such as the endocrine system, digestive system, and skin (5, 6). However, this patient developed a rare small intestinal sarcoma after receiving immunotherapy. There are no reports of immunotherapy causing new tumors, but we cannot deny that there is a relationship between immunotherapy and new tumors, as, during immunotherapy, the body's natural immune balance is interfered with. Furthermore, studies have found that ICIs are linked to epithelial–mesenchymal transition (EMT) and autophagy, which are associated with the development of malignant tumors (7).

## Conclusion

In this case, the patient was diagnosed with HCC 19 months before being diagnosed with intestinal sarcoma and achieved complete remission of HCC after treatment with PD-1 combined with bevacizumab. Based on the above information, we believe that this small bowel sarcoma was a rare complication of immunotherapy and should receive more attention.

## Data Availability

The original contributions presented in the study are included in the article/Supplementary Material, further inquiries can be directed to the corresponding authors.
